# Can social media data lead to earlier detection of drug‐related adverse events?

**DOI:** 10.1002/pds.4090

**Published:** 2016-09-07

**Authors:** Mei Sheng Duh, Pierre Cremieux, Marc Van Audenrode, Francis Vekeman, Paul Karner, Haimin Zhang, Paul Greenberg

**Affiliations:** ^1^ Analysis Group, Inc.BostonMAUSA; ^2^ Groupe d'analyse, LtéeMontréalQCCanada

**Keywords:** pharmacovigilance, adverse event, social media, Internet, Granger causality, pharmacoepidemiology

## Abstract

**Purpose:**

To compare the patient characteristics and the inter‐temporal reporting patterns of adverse events (AEs) for atorvastatin (Lipitor^®^) and sibutramine (Meridia^®^) in social media (AskaPatient.com) versus the FDA Adverse Event Reporting System (FAERS).

**Methods:**

We identified clinically important AEs associated with atorvastatin (muscle pain) and sibutramine (cardiovascular AEs), compared their patterns in social media postings versus FAERS and used Granger causality tests to assess whether social media postings were useful in forecasting FAERS reports.

**Results:**

We analyzed 998 and 270 social media postings between 2001 and 2014, 69 003 and 7383 FAERS reports between 1997 and 2014 for atorvastatin and sibutramine, respectively. Social media reporters were younger (atorvastatin: 53.9 vs. 64.0 years, *p* < 0.001; sibutramine: 36.8 vs. 43.8 years, *p* < 0.001). Social media reviews contained fewer serious AEs (atorvastatin, pain: 2.5% vs. 38.2%; sibutramine, cardiovascular issues: 7.9% vs. 63.0%; *p* < 0.001 for both) and concentrated on fewer types of AEs (proportion comprising the top 20 AEs: atorvastatin, 88.7% vs. 55.4%; sibutramine, 86.3% vs. 65.4%) compared with FAERS. While social media sibutramine reviews mentioning cardiac issues helped predict those in FAERS 11 months later (*p* < 0.001), social media atorvastatin reviews did not help predict FAERS reports.

**Conclusions:**

Social media AE reporters were younger and focused on less‐serious and fewer types of AEs than FAERS reporters. The potential for social media to provide earlier indications of AEs compared with FAERS is uncertain. Our findings highlight some of the promises and limitations of online social media versus conventional pharmacovigilance sources and the need for careful interpretation of the results. © 2016 The Authors. *Pharmacoepidemiology and Drug Safety* published by John Wiley & Sons Ltd.

## Introduction

Regulators have shown increasing interest in mining data from support group websites and social media postings as potential new sources for analyzing drug safety, patient‐reported outcomes, and drug use experiences.^1^, ^2^, ^3^ For example, the United States Food and Drug Administration (FDA) has stated that there is a public health interest in Internet‐based data and has funded the development of software to mine social media data for safety signals.^4^ Similarly, the Innovative Medicines Initiative, a public‐private partnership between the European Union and the pharmaceutical industry, recently funded a €6 million project to detect new drug‐related adverse events (AEs) by mining publicly‐available web and social media content.^5^

Leveraging social media data for pharmacovigilance requires an understanding of their strengths and weaknesses relative to conventional data sources, such as the FDA Adverse Event Reporting System (FAERS).^6^ However, the existing literature comparing social media and conventional pharmacovigilance data sources is limited to the comparison of the reporting characteristics without further investigating the inter‐temporal relationship of the reporting pattern between these sources.^7^, ^8^, ^9^ The objective of this study is to shed light on the potential role of social media data in pharmacovigilance, including their ability to accelerate the detection of drug‐related AEs. We compared the patient characteristics and patterns of AE reporting for two medications, atorvastatin (Lipitor^®^, a lipid‐lowering agent first approved in the USA in December 1996, manufactured by Pfizer, New York City, New York, United States) and sibutramine (Meridia^®^, a weight loss drug first approved in the USA in November 1997, manufactured by Abbott Laboratories, Abbott Park, Illinois, United States), in a social media data source with those in FDA FAERS reports.

## Methods

All statistical analyses were performed using stata (StataCorp LP, College Station, TX) version 14 or sas (SAS Institute, Inc., Cary, NC) version 9.3.

### Subjects

Atorvastatin (FDA approval December 1996) and sibutramine (FDA approval November 1997) were selected for this study to provide comparative evidence from the perspectives of a relatively safe versus an unsafe drug. Despite some known side effects, atorvastatin is generally viewed as having a favorable risk‐benefit profile.^10^ In contrast, the FDA withdrew sibutramine from the market in 2010 because of the occurrence of serious cardiovascular events, including heart attacks and strokes.^11^

### Data sources

#### Social media data source (AskaPatient.com)

AskaPatient.com is a patient support group website in which patients share and rate their experiences with different medications. We selected this social media data source because of the compatibility of this study with the website's terms of use.^12^

When creating a new review, users are required to enter basic demographic information (gender and age), medication dosage, duration and reason for use, and a satisfaction rating of 1 to 5, where 1 is defined as “I would not recommend taking this medicine” and 5 as “this medicine cured me or helped me a great deal.” In addition, users can enter free text in “side effects” and “comments” fields. We extracted data from these fields for reviews posted from 2001, when the website was established, to December 2014.

#### Conventional data source (FDA Adverse Event Reporting System reports)

FDA Adverse Event Reporting System is a database that contains information on adverse event and medication error reports submitted to FDA.^13^ We obtained all FAERS reports with “atorvastatin” or “sibutramine” in the generic preferred name field between November 1997 and December 2014 from DrugLogic (Reston, USA). Each report includes the following information: date received by the FDA, patient age and gender, and Medical Dictionary for Regulatory Activities (MedDRA) terms (i.e., System Organ Classes, High‐Level Group Terms, High Level Terms, and Preferred Terms) for the AEs included in the report, drugs listed on the report and their roles (e.g., “primary suspect drug,” “secondary suspect drug,” “concomitant,” or “interacting”), indicators for whether the AE was serious (i.e., resulted in death, hospitalization, disability, permanent injuries, or congenital anomaly or birth defect, or required intervention to prevent permanent impairment or damage^14^), and report source.

### Sample selection

We excluded social media reviews that: (i) provided insufficient information for use in our analyses (e.g., no information on type of side effects); (ii) contained only irrelevant, speculative, or hearsay information; and (iii) were obvious duplicates. To make the FAERS reports more comparable with the social media data, we excluded those with clinical trials or literature as their source (leaving reports from consumer and health professionals) and included only those in which atorvastatin or sibutramine was the primary suspect drug. We carried out sensitivity analyses including reports from clinical trials and literature.

### Classification of adverse events

We screened all social media reviews and categorized them into the following: (i) positive or negative overall sentiment; and (ii) AE categories. AE categories were generated by the reviewer during the screening process with the objective of capturing all user‐reported AE types and facilitating a reasonable degree of differentiation between AEs. The same social media review could contribute to multiple AE categories.

Our analyses also utilized specific, clinically important AEs for each drug (muscle pain and rhabodomyolysis for atorvastatin^15^ and cardiovascular AEs for sibutramine^11^). In the social media data, we identified pain from negative comments mentioning muscle, bone, or joint pain. Serious pain was identified from pain reports with comments consistent with the definition of a serious AE used by the FDA in the FAERS data.^14^ In the FAERS data, we identified pain using the MedDRA PTs listed in Appendix Table 1.^16^

In the social media data, we identified cardiovascular AEs based on reviews mentioning any cardiac‐related or vascular‐related issue. Serious cardiovascular AEs were identified by the same FDA criteria. In the FAERS data, cardiovascular AEs were identified using the MedDRA System Organ Classes “Cardiac Disorders” and “Vascular Disorders”.

### Comparisons of overall adverse event reporting patterns

We compared the gender distribution and mean age of AE reporters in the two data sources. Within social media reviews, we also compared the average user ratings for both drugs from all users and from the subset of users who experienced specific, clinically important AEs (user ratings were not available in FAERS). We presented *p*‐values of Student's *t*‐tests to assess the differences in means across the two data sources. Finally, we compared the frequencies of the most commonly mentioned AEs and specific AEs (overall and serious), the extent of overlap in the most commonly mentioned AEs, and the extent of heterogeneity in AEs (assessed by the fraction of unique reports that contribute to the top 10 and 20 AEs) between the two data sources. MedDRA High Level Term terms for AEs in FAERS were used for comparisons with the reviewer‐generated AE categories in the social media data; High‐Level Group Terms were used in a sensitivity analysis.

### Time series analysis and granger causality test

We used Granger causality tests to assess the association between the numbers of FAERS reports in a given month and social media postings in prior months, conditional on other available prior information (i.e., the number of FAERS reports in prior months). A variable*x*is said to “Granger‐cause” another variable*y*if *y* can be better‐predicted with all available prior information on both *x* and *y* than with prior information on *y* alone.^17^

Carrying out the Granger analyses first required us to model the relationship between FAERS reports and social media postings as a system of linear equations explaining the evolution of each variable as a function of the other variables in the system (i.e., a vector autoregressive model [VAR]). The VAR equations took the following form:

$$y_{t}=c+\sum_{i=1}^{T}\alpha_{i}y_{t-i}+\sum_{i-1}^{T}\beta_{i}x_{t-i}+u_{t}$$

We modeled the number of FAERS reports in a given month (*y*_*t*_) as a function of the numbers of FAERS reports in the prior months (*y*_*t* − *i*_) and prior social media postings (*x*_*t* − *i*_) in each of *T* prior months. As sensitivity analyses, we also modeled the proportional reporting ratio (PRR) for muscle pain and cardiac issues in FAERS in a given month (*y*_*t*_) as a function of the PRR for muscle pain and cardiac issues in FAERS in the prior months (*y*_*t* − *i*_) and prior social media postings (*x*_*t* − *i*_) in each of *T* prior months.

For each drug, we specified one VAR based on all AEs and one VAR based on specific AEs only. Our rationale for modeling both the overall number of reports and the number of reports for specific AEs was to explore the overall sensitivity of the predictive power (if any) of social media postings and its potential linkage to specific AEs.

For each VAR, we used likelihood‐ratio tests based on maximum lag lengths of up to 12 months to determine the optimal lag length *P*. We then executed the Granger causality tests on the equation representing the number of FAERS reports in a given month as a function of the numbers of past FAERS reports and social media postings in each of one through *P* prior months. This amounted to testing the null hypothesis of *β*_1_ = *β*_2_ = ⋅ ⋅ ⋅ = *β*_*p*_ = 0 in the previous equation, with *T = p*. When a two‐sided *p*‐value is 0.05 or less, we conclude that series *x_t_* Granger cause series *y_t_*.

## Results

### Sample selection

There were 998 social media postings for atorvastatin from April 2001 to December 2014 and 270 postings for sibutramine from June 2001 to October 2012. Our final FAERS dataset contained 69 003 atorvastatin from November 1997 to December 2014 and 7 383 sibutramine reports from March 1998 to May 2014.

### Summary statistics—atorvastatin

Table 1 presents the summary statistics for atorvastatin by data source. Similar proportions of social media postings and FAERS reports came from female reporters (48.2% and 53.3%, *p* = 0.096). Social media reporters were on average younger than FAERS patients (mean age 53.9 vs. 64.0 years, *p* < 0.001).

**Table 1:** Summary statistics for atorvastatin

	AskaPatient.com	FAERS	*p*‐value
Time period	2001 April to 2014 December	1997 November to 2014 December	—
Observations, *N*	998	69 003	—
Female, *N* (%)*	474 (48.2%)	32 898 (53.3%)	0.096
Age, mean (SD)	53.9 (10.8)	64.0 (12.7)	<0.001
Pain, *N* (%)†	670 (67.1%)	10 455 (15.2%)	<0.001
Serious pain, *N* (%)‡	17 (2.5%)	3996 (38.2%)	<0.001
Rating, mean (SD)			
Overall	2.1 (1.4)	—	—
Pain	1.7 (1.1)#	—	—
Serious pain	1.3 (0.6)§	—	—

AE, adverse event; FAERS, FDA Adverse Event Reporting System.

* Not available for all reviews/reports.

† Pain, AskaPatient.com: includes all comments related to muscle, bone, or joint pain; FAERS: includes AEs with Preferred Term for pain.

‡ AskaPatient.com: includes all comments mentioning life‐threatening AEs, hospitalization, disability or permanent damage, and AEs requiring intervention to prevent permanent impairment or damage; FAERS: includes AEs with Preferred Term for pain, and with “serious” drug outcome.

# *p*‐value versus pain <0.001.

§ *p*‐value versus serious pain = 0.136.

A larger proportion of social media reviews were related to pain compared with FAERS reports (67.1% vs. 15.2%, *p* < 0.001). The pattern is reversed for the subset of pain‐related AEs classified as serious: only 2.5% of pain‐related social media reports were serious compared with 38.2% of pain‐related FAERS reports (*p* < 0.001).

Three positive sentiment categories (effectiveness, no side effects, and cost covered by insurance) were identified among social media reviews of atorvastatin. Compared with all social media atorvastatin reporters, those who reported pain gave atorvastatin lower average ratings (1.7 vs. 2.1, *p* < 0.001); those who reported serious pain tended to give even lower ratings.

### Summary statistics—sibutramine

Table 2 presents summary statistics for sibutramine by data source. The gender distribution of reporters was similar across the two data sources (88.5% of social media reporters were female vs. 86.0% of FAERS reporters, *p* = 0.884), and social media reporters were on average younger than FAERS reporters (mean age 36.8 vs. 43.8 years, *p* < 0.001).

**Table 2:** Summary statistics for sibutramine

	AskaPatient.com	FAERS	*p*‐value
Time period	2001 June to 2012 October	1998 March to 2014 May	—
Observations, *N*	270	7383	—
Female, *N* (%)*	238 (88.5%)	6093 (86.0%)	0.884
Age, mean (SD)	36.8 (10.1)	43.8 (13.5)	<0.001
CV AEs, *N* (%)†	38 (14.1%)	1301 (17.6%)	0.160
Serious CV AEs, *N* (%)‡	3 (7.9%)	820 (63.0%)	<0.001
Rating, mean (SD)			
Overall	4.0 (1.1)	—	—
CV AEs	3.5 (1.4)#	—	—
Serious CV AEs	1.7 (1.2)§	—	—

AE, adverse event; CV, cardiovascular; FAERS, FDA Adverse Event Reporting System

* Not available for all reviews/reports.

† Cardiovascular issues, AskaPatient.com: includes all cardiac‐related or stroke‐related comments; FAERS: includes all AEs under the System Organ Classes “Cardiac Disorders” and “Vascular Disorders”, and additional Preferred Terms related to stroke.

‡ AskaPatient.com: includes all comments mentioning life‐threatening AEs, hospitalization, disability or permanent damage, and AEs requiring intervention to prevent permanent impairment or damage; FAERS: includes AEs with “serious” drug outcome.

# *p*‐value versus Overall = 0.012.

§ *p*‐value versus Serious CV AEs = 0.037.

The share of reports related to cardiovascular issues was similar across the two data sources (14.1% in AskaPatient.com vs. 17.6% in FAERS, *p* = 0.160). However, a smaller fraction of cardiovascular‐related social media reports were serious compared with cardiovascular‐related FAERS reports (7.9% vs. 63.0%, *p* < 0.001).

Compared with all social media sibutramine reporters, those who reported any cardiovascular issues gave sibutramine lower average ratings (3.5 vs. 4.0, *p* = 0.012), and those who reported serious cardiovascular issues tended to rate sibutramine even lower.

Six positive sentiment categories (weight loss, satiation, increased energy, increased motivation for diet or exercise, less irritability or anxiety, and antidepressant effect) were identified among social media sibutramine reviews. Consistent with the larger number of positive sentiment categories identified for sibutramine than for atorvastatin, the average rating for sibutramine in AskaPatient.com was higher than for atorvastatin (4.0 vs. 2.1, *p* < 0.001). This finding contradicted the clinical understanding of the efficacy and safety profiles of the two drugs: atorvastatin is generally considered efficacious and safe in the clinical community, and yet, patients gave it a lower average rating than sibutramine, which was withdrawn from the market by the FDA.

### Comparisons of adverse events across data sources

A total of 48 AE categories were identified among social media reviews for atorvastatin. Table 3 presents the top 20 AEs by data source. Muscle or bone pain and joint pain were the two most commonly mentioned AEs reported social media. Similarly, in FAERS, muscle pain (rank 1) and joint pain (i.e., musculoskeletal and connective tissue pain and discomfort; rank 3) were among the most common AEs reported. Several other top 20 AEs were common to both data sources.

**Table 3:** Comparison of the top 20 AEs for atorvastatin in AskaPatient.com and FAERS

	AskaPatient.com*		FAERS†	
	AE category (classified by reviewer)	%	AE category (MedDRA High Level Term)	%
1	Muscle or bone pain	58.3	Muscle pains	12.4
2	Joint pain	28.1	Asthenic conditions	11.2
3	Low energy	26.4	Musculoskeletal and connective tissue pain and discomfort	9.8
4	Mental fog or memory loss	18.8	Diabetes Mellitus (incl subtypes)	9.4
5	Cramps	15.7	Liver function analyses	8.0
6	Stomach or bowel issues	12.4	Muscle related signs and symptoms NEC	7.4
7	Weakness	11.2	Pain and discomfort NEC	6.9
8	Depression	10.4	Cholesterol analyses	5.6
9	Insomnia	10.3	Joint related signs and symptoms	5.4
10	Numbness	9.0	Myopathies‡	5.3
11	Headache	7.6	General signs and symptoms NEC	5.0
12	Vertigo	7.5	Skeletal and cardiac muscle analyses	4.3
13	Heart issue or chest pain	6.3	Gait disturbances	4.1
14	Blurry vision	5.8	Ischaemic coronary artery disorders	4.1
15	Swelling	4.9	Muscle weakness conditions	4.0
16	Skin issue	3.8	Physical examination procedures and organ system status#	3.8
17	Anxiety	3.5	Therapeutic and nontherapeutic responses§	3.5
18	Urinary issue	3.5	Paraesthesias and dysaesthesias	3.4
19	Flu symptoms	3.3	Death and sudden death	3.4
20	Mood swings	3.3	Nausea and vomiting symptoms	3.2

AE, adverse event; NEC, not elsewhere classified; FAERS, FDA Adverse Event Reporting System.

* AskaPatient.com: Negative side effects as classified by reviewer. “Pain” in Table 1 consists of Muscle or bone pain, Joint pain, Cramps, Headache, Heart issue or chest pain, and additional categories outside of the top 20.

† FAERS: High Level Term AE categories. “Pain” in Table 1 consists of selected Preferred Terms within the high level terms Muscle Pains, Musculoskeletal and connective tissue pain and discomfort, and Pain and discomfort NEC, and additional high level terms outside the top 20.

‡ Myopathies: consists of 9 Preferred Terms; top 3: “rhabdomyolysis”, “myopathy”, and “muscle necrosis“.

# Physical examination procedures and organ system status: consists of 26 Preferred Terms; top 3: “weight decreased”, “weight increased”, and “body height decreased”.

§ Therapeutic and nontherapeutic responses: consists of 23 Preferred Terms; top 3: “drug ineffective”, “drug effect decreased”, and “drug intolerance”.

A total of 18 AE categories were identified among social media reviews for sibutramine. Table 4 lists these AE categories as well as the 20 most‐common high level terms in FAERS. Dry mouth, headaches, and insomnia were the top 3 AEs mentioned in the social media reviews. As with atorvastatin, the top AEs in FAERS overlap substantially with those in AskaPatient.com.

**Table 4:** Comparison of the top 20 AEs for sibutramine in AskaPatient.com and FAERS

	AskaPatient.com*		FAERS†	
	AE category (classified by reviewer)	%	AE category (MedDRA High Level Term)	%
1	Dry mouth	37.4	Therapeutic and nontherapeutic responses‡	11.4
2	Headaches	35.6	Appetite disorders	11.0
3	Insomnia	30.7	Headaches NEC	9.8
4	Constipation	16.3	Disturbances in initiating and maintaining sleep	8.6
5	Cardiac symptoms	14.1	General signs and symptoms NEC	7.3
6	Anxiety or irritability	14.1	Asthenic conditions	7.1
7	Lack of efficacy	7.4	Vascular tests NEC (incl blood pressure)#	6.9
8	Nausea or indigestion	7.0	Nausea and vomiting symptoms	6.4
9	Excessive thirst	5.9	Anxiety symptoms	6.3
10	Low energy	5.6	Oral dryness and saliva altered	5.9
11	Muscle or joint pain	5.6	Neurological signs and symptoms NEC	5.8
12	Hypertension	4.8	Feelings and sensations NEC	5.6
13	Bad breath	3.0	Gastrointestinal atonic and hypomotility disorders NEC	5.3
14	Depression	2.6	Disturbances in consciousness NEC	5.2
15	Sexual dysfunction	2.6	Pain and discomfort NEC	4.9
16	Sweating	1.9	Breathing abnormalities	4.4
17	Skin lesions	1.5	Cardiac signs and symptoms NEC	4.1
18	Shortness of breath	0.7	Physical examination procedures and organ system status§	3.7
19	–	–	Heart rate and pulse investigations**	3.5
20	–	–	Paraesthesias and dysaesthesias	3.3

AE, adverse event; CV, cardiovascular; High Level Term, High Level Term; NEC, not elsewhere classified; FAERS, FDA Adverse Event Reporting System.

* AskaPatient.com: CV AEs in Table 2 consists of Cardiac symptoms.

† FAERS: High Level Term AE categories. CV AEs in Table 2 consists of the High Level Term Cardiac signs and symptoms NEC and additional High level terms and selected Preferred Terms related to stroke outside the top 20.

‡ Therapeutic and nontherapeutic responses: consists of 12 Preferred Terms; top 3: “drug ineffective”, “therapeutic response unexpected”, and “drug effect decreased”.

# Vascular tests NEC: consists of 8 Preferred Terms; top 3: “blood pressure increased”, “blood pressure decreased”, and “blood pressure systolic increased”.

§ Physical examination procedures and organ system status: consists of 12 Preferred Terms; top 2: “weight increased”, and “weight decreased”.

** Heart rate and pulse investigations: consists of 7 Preferred Terms; top 3: “heart rate increased”, “heart rate irregular“, and “pulse absent”.

The frequencies of the top AEs listed in Tables 3 and 4 suggest that, compared with FAERS, social media reviews tended to concentrate on the same few AEs. For atorvastatin, the top 20 AEs comprised 88.7% of social media reviews compared with 55.4% of FAERS reports. There was also less variety among social media reviews for sibutramine compared with FAERS, where the top 20 AEs comprised almost all social media reviews versus less than two‐thirds of FAERS reports. Results were similar when we used MedDRA High‐Level Group Terms (i.e., a higher level of aggregation) instead of High Level Terms to classify AEs in the FAERS data, indicating that the greater heterogeneity among FAERS reports was not an artifact of particular AE aggregations. Including reports from clinical trials and literature does not have any substantial impact on any of the summary statistics or AE reporting patterns.

### Time series analysis

**Figure 1:**
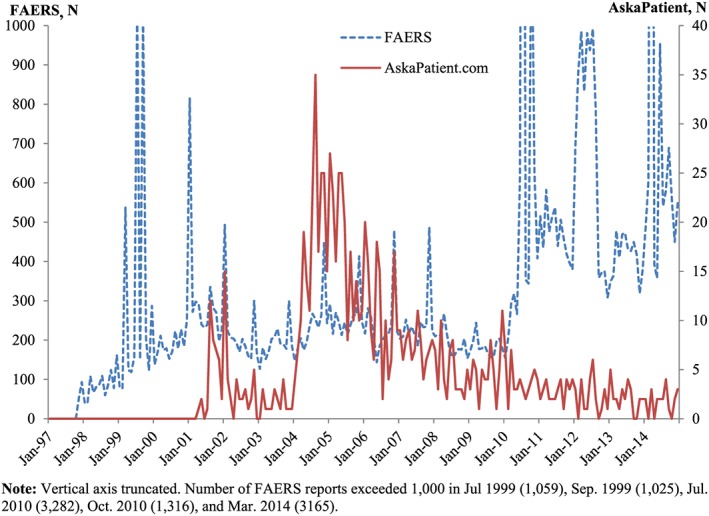
Time series of FDA Adverse Event Reporting System (FAERS) reports and social media postings for atorvastatin

**Figure 2:**
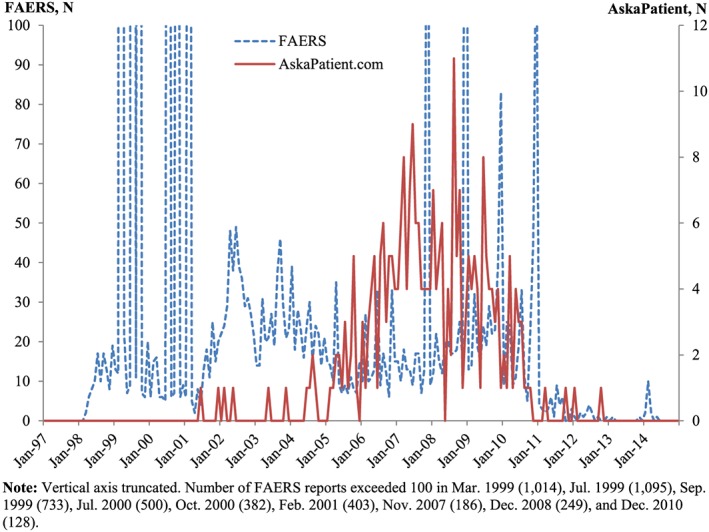
Time series of FDA Adverse Event Reporting System (FAERS) reports and social media postings for sibutramine

Figures 1 and 2 depict time series of social media postings and FAERS reports for atorvastatin and sibutramine. FAERS reports spike soon after the launches of both drugs. For atorvastatin, there is no clear relationship between the social media and FAERS reporting patterns. For sibutramine, despite greater volatility due to the smaller total numbers of reports compared with atorvastatin, the pattern is clearer: spikes in social media postings seem to predate those in FAERS, especially between 2007 and 2011.

Table 5 presents results of Granger causality tests of the inter‐temporal relationship between social media postings, FAERS reports. For atorvastatin, likelihood‐ratio tests indicated an optimal lag length of 3 months for the all‐reports equation. The Granger test results were consistent across all atorvastatin VARs. there is no evidence that social media postings help predict FAERS reports.

**Table 5:** Granger causality tests: do social media postings help predict subsequent FAERS reports?

Drug	AEs	Granger test results* (indep. variable = No. of AskaPatient.com postings)			
Dep. variable = No. of FAERS reports		Sample size (months)	Lag length (months)†	χ^2^	*p*‐value
Atorvastatin	All	128	3	1.36	0.715
	Muscle pain	128	3	0.86	0.836
Sibutramine	All	55	4	24.17	<0.001
	Cardiac issues‡	35	11	57.90	<0.001

AE, adverse event; CV, cardiovascular; FAERS, FDA Adverse Event Reporting System.

* Null hypothesis: AskaPatient.com reports do not Granger‐cause FAERS reports.

† Specified lag length was optimal based on results of likelihood‐ratio tests for all vector autoregressions of order ≤12 months.

‡ Analyses of CV AEs focused on cardiac issues because AskaPatient.com reviews mentioning CV AEs did not mention any vascular events.

For sibutramine, social media reviews mentioning cardiovascular events did not mention vascular events. Hence, our analyses of the time series of specific AEs excluded vascular events from the FAERS data. The optimal lag length for the all‐reports equation was 4 months. The total number of social media postings Granger‐cause FAERS reports 4 months later (*p* < 0.001). The predictive power of social media postings for sibutramine appears to be at least in‐part explained by social media reviews mentioning cardiac‐related AEs, which Granger‐cause FAERS reports for cardiac issues (optimal lag length = 11 months; *p* < 0.001). Sensitivity analyses using PRRs for muscle pain and cardiac issues showed similar pattern for both atorvastatin and sibutramine. Including reports from clinical trials and literature does not have any substantial impact on the Granger causality analyses.

## Discussion

The findings of this study highlight some of the promises of social media data sources for detecting early AE reports patterns compared with conventional pharmacovigilance tools. Social media AE reports helped predict the occurrence of FAERS reports several months later for only one out of the two medications we studied. A possible explanation for these mixed results is that sibutramine reporters tended to be younger (average age in AskaPatient.com = 36.8 years vs. 53.9 years for atorvastatin). To the extent that overall social media utilization (and, as a byproduct, AE reporting rates in online health forums) are higher for younger patients, the drivers of social media AE reports over time may tend to mirror those for conventional pharmacovigilance sources to a greater extent for medications utilized more frequently by younger patients.

In addition, despite some similarities between the two data sources (e.g., top AEs), our results suggest that social media sources contain different information and are used by a different demographic group compared with conventional pharmacovigilance data sources such as FAERS. Compared with FAERS, social media reporters of drug‐related AEs tended to be younger, and their reviews focused on fewer and less‐serious AEs affecting their quality of life rather than clinically‐significant AEs. These results were consistent across two drugs with very different safety profiles. Moreover, our results are consistent with the conclusions from the literature. Pages *et al*.^8^ found that AEs associated with oral antineoplastic drugs reported in online forums were less serious compared with what is reported in the French pharmacovigilance database. Similarly, in a systematic review of studies assessing the prevalence and type of information on AEs in social media, Golder *et al*.^9^ found that social media have higher report frequency on symptom‐related and mild AEs.

Context provided in social media reviews was important to our study. For example, social media reviews expressed both positive and negative sentiments, and some postings in the raw data appeared to be based on hearsay rather than actual experiences by the social media reviewer. Our manual review process was well‐suited to identifying and appropriately interpreting this context. Freifeld *et al*. reached a similar conclusion in their 2014 study of the extent of concordance between Twitter posts mentioning AEs and FAERS reports, noting that “…a human curation step was the most efficient way to understand the nature of the problems reported.”^18^

Researchers and regulators should be attentive to potential differences in the information contained in social media versus conventional pharmacovigilance data sources. One policy implication of our findings, consistent with the conclusions of the Freifeld *et al*. study discussed previously, is that AE reports from social media sources should not be pooled together with those from conventional pharmacovigilance sources (e.g., by merging social media AE reports into the FAERS system). Clinically important signals that the current FAERS system is designed to capture could be diluted by the large influx of non‐life‐threatening AEs that seem to be prevalent in social media sources. Hence, it is important that pharmacovigilance regulations provide clear guidance about what constitutes a reportable event to ensure the quality of the data (e.g., hearsay reports of AEs should not be combined with direct reports by reporters who experienced AEs after taking a medication). In addition, regulators should maintain detailed data on the source of AE reports and make these data available to researchers.

This study has several limitations. First, the generalizability of our findings to other medications and data sources is uncertain. We focused on only two medications based on their divergent risk‐benefit profiles and hence abilities to provide different perspectives on social media versus conventional pharmacovigilance sources. Second, AskaPatient.com is only one social media site. The number of postings is small, which is likely to result in volatile time series. It may not be representative of all social media sources, such as open discussion forum or major social networks. Third, AskaPatient.com was established in 2000 and the first reports for atorvastatin and sibutramine were not available until 2001, several years after the approval of these two drugs. Also, the Internet use was not as prevalent. Because the majority of spontaneous reports usually occur during the first few years following the approval of a new drug, AskaPatient.com might not capture the full AE profiles of these two drugs. Hence, inferences from this study may not apply equally to other social media sources or to more recently approved drugs. Fourth, both FAERS and AskaPatient.com data consist of unvalidated patient self‐reports possibly containing errors and duplicates. Fifth, as with all spontaneous AE report sources, neither AskaPatient.com nor FAERS have information on the total number of users of a specific drug needed to calculate AE rates. Both are numerator‐based reports which may be subject to the influences of news media, herd effects, and hindsight bias. Sixth, because of the lack of data on patient characteristics, our Granger causality tests did not control for confounding factors that might affect the reporting patterns in each data source. Seventh, the total number of reports and changes in the number of reports over time are imperfect measures of a drug's potential safety issue. They not only contain true safety signals, but also noises that are subject to stimuli such as media reports, legal actions and FDA safety communications. Exploring and comparing the patterns of safety signals using appropriate metrics (e.g., PRR or EBGM scores) between these data sources is an important topic for future research.

**Key Points**

- Social media adverse event reporters were younger and focused on fewer and less‐serious adverse events compared with FAERS reporters.
- The potential for social media sources to give earlier indications of adverse events compared with FAERS is uncertain.
- Appropriate interpretation and methods of analyses of adverse events from various data sources are crucial to properly evaluate hypothesis‐generating drug safety signals.

## Conflict of Interest

The paper is self‐initiated by the authors, who are all employees of Analysis Group. No outside funding was provided. Analysis Group is a consulting company that has provided professional services to various government agencies and industries, including the pharmaceutical, computer IT, energy, and banking industries. The authors thank AskaPatient.com for providing their data for use in this study.

## Prior Postings and Presentations

Preliminary results of this study were presented at the International Conference on Pharmacoepidemiology (2014, 2015) and the Epidemiology Seminar Series at the Harvard T.H. Chan School of Public Health (2015).

## Appendix

**Table:** Table A1. Selected MedDRA preferred terms used to identify pain

Abdominal pain	Hernia pain	Perineal pain
Abdominal pain lower	Laryngeal pain	Phantom pain
Abdominal pain upper	Ligament pain	Pleuritic pain
Back pain	Lip pain	Psychogenic pain disorder
Bladder pain	Lymph node pain	Pubic pain
Bone pain	Musculoskeletal chest pain	Radicular pain
Breakthrough pain	Musculoskeletal pain	Renal pain
Breast pain	Myofascial pain syndrome	Salivary gland pain
Chest pain	Neck pain	Scrotal pain
Complex regional pain syndrome	Nipple pain	Spinal pain
Ear pain	Non‐cardiac chest pain	Suprapubic pain
External ear pain	Oesophageal pain	Tendon pain
Eye pain	Oral pain	Testicular pain
Eyelid pain	Oropharyngeal pain	Thyroid pain
Facial pain	Pain	Urethral pain
Flank pain	Pain in extremity	Urinary tract pain
Gallbladder pain	Pain in jaw	Uterine cervical pain
Gastrointestinal pain	Pain management	Uterine pain
Genital pain	Pain of skin	Vein pain
Gingival pain	Patellofemoral pain syndrome	Vulvovaginal pain
Groin pain	Pelvic pain	
Hepatic pain	Penile pain	
